# Transcranial direct current stimulation (tDCS) facilitates overall visual search response times but does not interact with visual search task factors

**DOI:** 10.1371/journal.pone.0194640

**Published:** 2018-03-20

**Authors:** Kyongje Sung, Barry Gordon

**Affiliations:** 1 Department of Neurology, The Johns Hopkins University School of Medicine, Baltimore, Maryland, United States of America; 2 Cognitive Science Department, The Johns Hopkins University, Baltimore, Maryland, United States of America; University Medical Center Goettingen, GERMANY

## Abstract

Whether transcranial direct current stimulation (tDCS) affects mental functions, and how any such effects arise from its neural effects, continue to be debated. We investigated whether tDCS applied over the visual cortex (Oz) with a vertex (Cz) reference might affect response times (RTs) in a visual search task. We also examined whether any significant tDCS effects would interact with task factors (target presence, discrimination difficulty, and stimulus brightness) that are known to selectively influence one or the other of the two information processing stages posited by current models of visual search. Based on additive factor logic, we expected that the pattern of interactions involving a significant tDCS effect could help us colocalize the tDCS effect to one (or both) of the processing stages. In Experiment 1 (n = 12), anodal tDCS improved RTs significantly; cathodal tDCS produced a nonsignificant trend toward improvement. However, there were no interactions between the anodal tDCS effect and target presence or discrimination difficulty. In Experiment 2 (n = 18), we manipulated stimulus brightness along with target presence and discrimination difficulty. Anodal and cathodal tDCS both produced significant improvements in RTs. Again, the tDCS effects did not interact with any of the task factors. In Experiment 3 (n = 16), electrodes were placed at Cz and on the upper arm, to test for a possible effect of incidental stimulation of the motor regions under Cz. No effect of tDCS on RTs was found. These findings strengthen the case for tDCS having real effects on cerebral information processing. However, these effects did not clearly arise from either of the two processing stages of the visual search process. We suggest that this is because tDCS has a DIFFUSE, pervasive action across the task-relevant neuroanatomical region(s), not a discrete effect in terms of information processing stages.

## Introduction

The possible effects of transcranial direct current stimulation (tDCS) on mental functions, and the physiological basis for any such effects, have been extensively investigated and strongly debated in recent years. There have been many reports that tDCS can positively affect almost all functions tested [1–5]. More specifically, in the case of visual processes in healthy controls, studies have indicated that tDCS can affect neurophysiological and behavioral responses, such as improving or impairing visual signal detection thresholds, altering visual evoked potentials, or affecting visual search performance [2, 3, 6–8]. However, some recent reviews of tDCS studies claim that the data have been overinterpreted or misinterpreted, and that tDCS actually has little or no effect on mental functions [9–11].

Perhaps because of this uncertainty about the tDCS effect, much of the focus of studies to date has been on establishing its existence. Less attention has been paid to investigating how any such effects on overt behaviors might arise from effects on underlying neurophysiological activities and to understanding such effects at the levels of human information processing. The experiments we report in this study attempt to address these issues: testing for significant and reliable tDCS effects and trying to localize the underlying neuroanatomy of tDCS effects and their origins in terms of information processing models.

### The visual search task

Our approach was to examine whether tDCS might affect performance in a task whose underlying theoretical mechanisms have been well understood in information processing terms, and for which some plausible neuroanatomical correlates of those information processing mechanisms have been proposed. We suggest that a visual search task that requires attentional engagement meets these requirements. In the current study, we employed a visual search task in which participants search for a single known target (“L”) among distractors (rotated “Ts”). Studies have shown that this type of search task generates increasing search response times (RTs) as the number of distractors increases (i.e., a significant set-size effect) [12–15], which is a behavioral index of attentional engagement.

When the stimuli are limited to central vision, there is general agreement that two distinct processing stages arranged in a sequence are responsible for search performance in this task [16–20]. In the first of these sequential stages, basic visual features (e.g., the presence of horizontal and vertical strokes that make up the stimulus “L”) are extracted from the stimuli, and the location maps of individual features (i.e., feature maps) are formed in parallel [18, 19]. This first stage does not require attention, according to these models. In the second stage, the individual feature maps are conjoined into a complete representation of the stimuli (termed a “master saliency map”). The target stimulus in memory (the “L”) is then sequentially compared against these representations of the stimuli in the saliency map. This second stage does require attention.

Several factors have been shown to affect RTs in this type of task [12, 13, 21–25], such as the visual quality of the stimuli and the number of distractor items. In particular, specific task factors have been shown to affect one or the other of the two processing stages. Altering the visual quality of the stimuli (e.g., diminished brightness, decreased contrast, added white noise, etc.) selectively influences the processes of the first stage, modulating the overall mean search RTs [21, 23–25]. Changing the number of distractor items and their similarity to each other (and to the target) selectively affects the second stage. A greater number of distractors and greater similarity lead to prolonged RTs [12, 13, 26]. Another factor affecting the second stage is whether or not the target item is present in the stimulus display. When a target is present, the mean RT is typically shorter than when a target is absent because the search for a target among possible candidates in the master saliency map is self-terminating [27].

### Identifying the site(s) of RT effects

The stage architecture of the processes involved in these forms of visual search, and the selectiveness of task factors in these processing stages, have been delineated using additive factor logic [28, 29]. According to this logic, within the scope of a serial-parallel network of processes [30], if two factors selectively influence the duration of two different processes of the network, and the two processes are arranged sequentially in the network, then the combined effect of the two factors on the overall duration of the network must be additive (no interaction). If the two processing stages are arranged in parallel (or concurrent, in network model terms), then the combined effect must not be additive (significant interaction).

In the type of visual search task discussed here, manipulations of visual quality (e.g., brightness, contrast, etc.) have been shown to have additive effects (no interactions) with manipulations of target-distractor similarity or the number of distractors [21, 22, 25]. However, the simultaneous manipulations of target-distractor similarities, the number of distractors, and the presence/absence of the target have been shown to have nonadditive effects (interact with each other) [12, 13, 25, 27]. These patterns of interaction effects on RTs are the evidentiary basis for the existence of at least two sequential processing stages in this visual search task [31–33]. The first stage is affected by stimulus brightness or contrast changes [21, 23–25]; the second stage, by target-distractor similarity, the number of distractors, or target presence/absence [12, 18, 19, 34]. The patterns of interactions of factors support the conclusion that these two processing stages are arranged sequentially [16, 18, 19]. While attentive processing in the second stage is the important part of the two-stage model for the visual search process, attentional engagement itself does not necessarily imply that the processes in the second stage operate in series. The serial processing assumption in some two-stage models can be easily modified [17]; parallel processing can also give rise to a set-size effect [12, 35]. However, this flexibility in process organization does not affect the conclusion based on the additive factor logic, because the interaction between factors affecting the same processes in the second stage would still occur whether their processing is serial or not.

In our current study, we used this detailed understanding of the theoretical processes underlying this visual search task, and of the factors that affect either processing stage in this task, as a tool to identify the locus of any effects of tDCS on a visual search task.

### Possible neuroanatomical associations

Studies on the plausible neuroanatomical substrate or substrates for the key theoretical components of the visual search process have focused on the location of the feature and saliency map of visual display with diverse properties of stimuli, including stimuli that comprise basic features (line segment or brightness contrast), geometric stimuli (junction of basic features), or complex stimuli such as natural scenes [36–48].

Studies on visual attentional control in nonhuman primates have suggested subcortical and cortical areas, such as pulvinar, superior colliculus, and visual cortex, as the possible locations of saliency maps of simple objects with basic features [36, 37, 39]. Many imaging and electrophysiological studies with humans have offered evidence for the saliency map of basic and complex stimuli in structures along the visual pathway and part of the parietal cortices [39–44], which have a strong association with the subcortical structures reported in animal studies.

Importantly, this evidence suggests that the hierarchical nature of visual information processing along the visual pathway may create different saliency maps distributed across relevant regions of the brain, depending on the level of abstraction of visual information [45–48]. For example, the lateral geniculate nucleus may already form a certain bottom-up saliency map for luminance contrast or color of stimuli without any object or feature identity information. Later in visual processing, different areas of the visual cortex may form saliency maps of stimuli, such as orientation and translation of a line or dot and conjunction of them (e.g., geometric shapes and patterns) in primary and adjacent visual areas [43, 45, 48, 49]. It appears that the saliency map for a more complex scene (e.g., natural scenes) is dependent on a wide range of brain structures, including a part of the posterior cortex [41, 45, 48, 50].

Given these findings on the possible neuroanatomical regions associated with the saliency map of visual stimuli at different levels of processing, the visual cortex seems to be the cortical region that represents the stimulus-driven feature and saliency maps of visual representations of stimuli (basic features and geometric shapes/patterns). These maps are, in turn, thought to be modulated recursively via top-down endogenous attentional control associated with the right posterior parietal cortex (rPPC) [47, 51, 52].

### Implications for probing the possible effects of tDCS

These lines of evidence suggest that applying tDCS to the visual cortex and surrounding regions could possibly affect the neuronal processes associated with the stages involved in the visual search task. If we observe a significant main effect of tDCS on search RTs, then the patterns of interaction between tDCS and the task factors that are known to affect RTs would reveal the locus of these tDCS effects.

We expected several results based on previous reports of tDCS effects. Prior studies [7, 8] have found that tDCS could have a significant effect on behavioral responses (e.g., RT and response accuracy) in visual search tasks, suggesting that we would also find an effect of tDCS on visual search RTs. We also had expectations about what different polarities of stimulation would do. It has been suggested that anodal tDCS operates by enhancing the signal/noise (S/N) ratio of early visual input in the visual cortex, whereas cathodal tDCS degrades the S/N ratio [1–3]. If that is the case, then we would expect that anodal tDCS over the responsible visual areas would improve overall search RTs by enhancing the S/N ratio of the feature representations in the feature maps or by increasing the ratio of the activation levels between targets and distractors in the stimulus-driven master saliency map. Conversely, cathodal tDCS over these areas would be expected to prolong overall search RTs through the reverse mechanisms.

### Other design considerations

Because of the criticisms and concerns that have arisen about studies using tDCS, and about psychological experiments in general, our study was designed with several safeguards.

One safeguard was to try to control for possible carryover effects. There is evidence of possible carryover effects between tDCS sessions that may persist even after several days [53, 54]. For practical reasons, we could not have participants wait for long periods of time between sessions to eliminate any conceivable carryover effect. However, we did ensure that the three experimental sessions (sham/anodal/cathodal) were conducted on three different days, with at least one full day between test sessions. We also took steps to control for participants’ schedules, which caused the length of the washout period between sessions to vary (see “Experimental procedure” for Experiment 1). To negate these varying-length washout periods, we included an additional sham condition in each experimental session so that we could statistically adjust for any plausible confounding effects between sessions.

There have also been concerns about whether subjects can be completely blinded to the presence or absence of electrical stimulation during tDCS [55–57]. For the tDCS administration protocol and equipment we used, there is no agreed-upon methodology for preventing awareness of stimulation. In fact, our pilot testing showed that some participants were indeed able to identify active electrical tDCS stimulation, especially when it immediately followed a sham condition. This also proved to be true in the main experiments for some participants. Therefore, in our experiments, we asked participants to rate their subjective awareness of whether tDCS was applied or not, and we analyzed these data to determine whether such awareness might have affected the results.

We chose to stimulate simultaneously with performance of the task (“online”) rather than before the task (“offline”). This choice was made because previous tDCS studies on visual processes have shown that the effect of tDCS on behavioral measures seems to be maximized with online stimulation [1, 3, 7, 8, 58, 59]. Therefore, we chose to apply tDCS online both to maximize the expected effect size and to maximize compatibility between our tDCS protocol and prior protocols specifically focused on visual search task performance.

We were also aware of important concerns about the replicability of tDCS effects on behavioral measures of perceptual and higher cognitive functions [9–11, 60], and about the replicability of psychological experiments in general [61, 62]. We did our own replication, duplicating the conditions of the first experiment within a more comprehensive second experiment with a larger sample.

Lastly, we added an experiment (Experiment 3) to help eliminate concerns about a possible anatomical confound. The electrode placement montage of Experiments 1 and 2 included the vertex (Cz) electrode and the electrode at Oz. Therefore, it was conceivable that the RTs obtained were affected by incidental stimulation of motor cortex subjacent to Cz. We evaluated this possibility in Experiment 3 by having one electrode placed over Cz and the other electrode over an extracephalic location (right upper arm).

## Experiment 1

### Materials and methods

#### Rationale and design

In Experiment 1, we manipulated two of the factors known to affect the attentive stage of the two-stage model of visual search (target-distractor discrimination difficulty and target presence/absence) to see if they interacted with any effects of tDCS. There were three levels of task difficulty and two levels of target factors in a repeated-measures design.

To detect any tDCS effects, we administered two identical visual search tests (Test 1 and Test 2) in each of three tDCS sessions. In each session, Test 1 always served as a sham, and Test 2 served as either another sham, anodal stimulation, or cathodal stimulation; these were referred to as sham-sham (S-S), sham-anodal (S-A), and sham-cathodal (S-C) sessions, respectively. We tested participants in one of the counterbalanced orders for these three sessions. Therefore, the experimental design of each session was a 3 (discrimination difficulty) × 2 (target presence/absence) × 2 (test-set) repeated-measures factorial design. Each session was analyzed separately (see “Statistical analysis”). In this design, the statistical significance of the test-set (Test 1 and Test 2) factor would indicate either a significant tDCS effect or no effect in each active stimulation session. Testing the effect of tDCS with the test-set factor (i.e., the difference between Tests 1 and 2 in a session) would help us statistically nullify any carryover effects from an active tDCS session (anode or cathode) to a subsequent session, given the reasonable assumption that the carryover effect was present for all the tests in the subsequent session.

#### Participants

By the end of Experiment 1, a total of 15 healthy college undergraduates had been recruited to participate in the study. We had originally aimed to recruit 12 participants to randomly assign two participants to each of the six possible counterbalanced session orders. However, we discovered that two participants in the initial group did not follow the experimental instructions and produced excessive errors (see “Data cleaning”). Another participant could not finish all three required experimental sessions. Therefore, we excluded the data from these three subjects from the study and recruited three additional participants to achieve an equal number of participants in each session. Of the 12 subjects included in the study, four were women. All subjects reported normal or corrected-to-normal vision, and all were right-handed. The mean (SD) of ages was 22.8 (1.9) years.

All participants gave written informed consent to participate. During the consenting process, participants were informed that they might receive electrical stimulation during the three experimental sessions, but they were not informed of when they might get such stimulation (making this a single-blind experimental design).

All participants were paid for their participation. The study and consent procedures were approved by the Johns Hopkins Medicine Institutional Review Board.

#### Visual search task, apparatus, and stimuli

**Fig 1:**
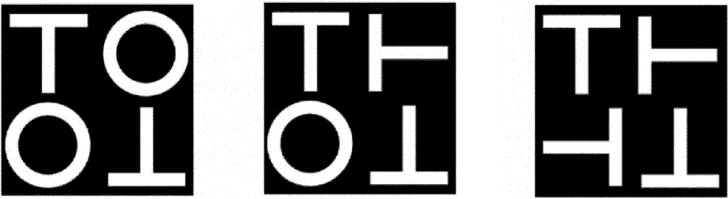
Experiment 1: Three examples of target-absent condition display. Left: Easy search. Middle: Intermediate search. Right: Difficult search. In the target-present condition, the “L” replaced the upright “T” or the “T” rotated 180°.

We adapted and modified a visual search task (Fig 1) from previous studies [12, 13]. The search task was to find an “L” among “Os” and “Ts” that were upright or rotated 90°, 180°, or 270°. As noted earlier, studies have demonstrated that this particular task generates a significant set-size effect and requires explicit attentional engagement [13–15].

Participants were instructed to press the “J” key of a computer keyboard with their right index finger if they found a target, and to press the “F” key with their left index finger if they did not find a target. They were told to keep their two index fingers positioned on the response keys at all times and to respond as quickly as possible. Fig 1 shows three examples of the stimulus display without the target upright “L” (target-absent conditions). We manipulated the discrimination difficulty by changing the number of rotated “Ts” in the stimulus displays [12, 13]. There were three task difficulties: easy, intermediate, and difficult. For the easy condition, we presented an upright “T,” a “T” rotated 180°, and two “Os” (Fig 1: left). For the intermediate condition, we replaced one “O” from the easy condition with a “T” rotated either 90° or 270° (Fig 1: middle). For the difficult condition, we replaced both “Os” from the easy condition with “Ts” rotated 90° and 270° (Fig 1: right). Thus, when the target did not appear, an upright “T” and a “T” rotated 180° were always presented. When the target “L” appeared, it replaced the upright “T” or the “T” rotated 180° an equal number of times.

One merit of the discrimination difficulty manipulation described above was that the difficulty manipulation is equivalent to the set-size manipulation in a typical visual search experiment [12]. Assuming that the “Os” are effectively inhibited by the attentional system [12, 19], or rotated “Ts” receive a higher priority for being attended to than “Os” due to their similarity to the target “L” [16, 17], replacing “Os” with rotated “Ts” would produce an effect similar to the typical set-size effect. That is, the mean RT would increase as the number of rotated “Ts” increased in the search display. Therefore, the interpretation of the results from the manipulation of discrimination difficulty could be applied to the results from the manipulation of set size.

The size of each stimulus was 46×46 pixels. We arranged the stimuli randomly in imaginary 2×2 cells with a four-pixel gap between stimuli. The size of a stimulus display with four stimuli was thus 96×96 pixels, which corresponded to approximately 2.6° of visual angle on a 1280×1024-pixel LCD monitor (37.5×30 cm of the viewable area with a 60-Hz refresh rate) from a viewing distance of about 50 cm. No chin rest was used. White stimuli (luminance of 135 cd/m^2^) appeared on a black background (0.3 cd/m^2^) in a dimmed room. The stimuli presentation and data acquisition were done with E-prime software (Psychology Software Tools, Inc.; version 2.0) on a Windows XP PC (Optiplex 745; Dell, Inc.).

#### Direct current stimulation

We placed saline-soaked sponge electrodes (5×7 cm) over Oz and Cz, according to the international 10/20 electroencephalogram system. Following convention, the polarity of current applied to the electrode over Oz determined whether the tDCS stimulation session was termed “anodal” (when the polarity was positive) or “cathodal” (when the polarity was negative). A current of 1.5 mA was delivered through the sponge electrodes using a 9-V battery-driven constant current stimulator (Chattanooga Ionto^™^ Dual Channel Iontophoresis System). At peak current (1.5 mA), the current density of the stimulation electrodes was 0.0429 mA/cm^2^. For each sham stimulation, the experimenter ramped current up from 0 mA to 1.5 mA over a span of approximately 30 seconds, starting at the beginning of the trial, and then ramped it down to 0 mA over another approximately 30 seconds.

#### Experimental procedure

We tested participants in three sessions—S-S, S-A, and S-C—on three different days with at least one full day of washout period (not exceeding 7 days; the mode was 2 days) between sessions. Each session lasted about 50 minutes with a 10-minute break between the two search tests, Test 1 and Test 2.

At the beginning of each session, the experimenter put the sponge electrodes on the participant’s head. The experiment started with eight practice trials. Then four blocks of experimental trials, with 96 trials in each block, were conducted in each of the two visual search tests. We randomly presented all possible combinations of experimental factors (target presence and task difficulty) in a block of trials. Therefore, four blocks of trials in a test were identical except for the random order of trial presentation in each block.

In each trial, a fixation cross appeared for 1 second in the middle of the screen. A set of stimuli was presented immediately after the fixation cross disappeared and remained on the screen until a participant pressed a key. If no response was made for 10 seconds, the stimuli disappeared, and the next trial started. Participants were allowed to take a self-paced break (not exceeding 2 minutes) between blocks of trials. Participants took approximately 15 minutes, but not more than 20 minutes, to complete each visual search test. The duration of active tDCS during Test 2 (S-A and S-C sessions) varied slightly across participants but never exceeded 20 minutes.

At the end of each search test (Tests 1 and 2), the experimenter gave participants two questionnaires: one about possible side effects they might have experienced and the other about their subjective awareness of electrical stimulation. For the latter, participants rated their awareness of stimulation on a scale ranging from 0 to 10 (0 = “extremely confident that I was not receiving electrical stimulation”; 5 = “not sure”; 10 = “extremely confident that I was receiving electrical stimulation”).

### Analysis

#### Data cleaning

As noted earlier, two participants recruited initially were excluded from the study because they did not seem to follow the instructions for making speeded responses: one produced 44% of outliers and errors in the S-A session, and the other produced 21% of outliers and errors in the S-S session. These error rates were considered excessive given the overall error and outlier rates (see below). We therefore replaced the data from these two participants with the data from two new participants.

On average, 12 participants included in the data analysis had an erroneous response rate of 3.8% (false alarms and misses; 1,055 trials); these erroneous trials were removed before we defined and removed RT outliers. RTs were identified as outliers using the following criteria: For each combination of factors in the six visual search tests completed by participants, RTs were considered to be outliers if they were longer than 1 second or 3 SDs above the mean RT for the condition or less than 250 ms [12]. These rules excluded 2.3% (607) of responses from the total number of correct responses.

#### Statistical analysis of RTs

For statistical analysis, the average RTs of each combination of factor levels for each participant were calculated for each session. We then conducted a repeated-measures analysis of variance (ANOVA) on the average RTs of the 12 participants for each session separately with a Bonferroni correction for multiple comparisons. Thus, the statistical significance was tested at an alpha level of .017 (.05/3).

#### Analysis of subjective ratings

Each participant provided a total of six ratings of perceived electrical stimulation: one rating after each search test (Test 1 and Test 2) in the three sessions. The distributions of these ratings were often extremely skewed or nonunimodal (see S1 and S2 Tables). Therefore, we determined that conventional statistical tests, such as ANOVA or the *t* test, were not appropriate for these rating data. Instead, we calculated the descriptive measures (mean and SD) of the subjective ratings and visually examined their relationship with any observed tDCS effects in two separate procedures.

To see if participants were blind to stimulation, we first identified those who made false alarms on the electrical stimulation during the S-S session with high confidence (rating ≥7) and examined whether any characteristics of search performance or experimental conditions led to their false judgment. Also, we identified those who correctly judged the S-S session as a sham and examined whether they could judge the true electrical stimulations (the S-A and S-C sessions) with high confidence as well.

Then, to see if any observed tDCS effects might be attributable to participants’ subjective appreciation of the electrical stimulation, we split the participants into two or three groups by the session in which they produced the largest RT change between Test 1 and Test 2. Then, the RT changes in these subgroups were compared with participants’ ratings to see if the subjective ratings matched their search performance, regardless of their false alarms in the S-S session. Any positive associations between search performance and subjective ratings would indicate that the observed effect of tDCS, if any, was confounded with participants’ apparent awareness of electrical stimulation.

### Results

#### Mean RTs and ANOVA

Table 1 lists mean (SD) of search RTs of all combinations of the three experimental factors—discrimination difficulty, target presence, and test set—for each session. The test of the equality of variance (Mauchly’s test) showed no violation of sphericity assumption for covariance matrix except for the test of the main effect of difficulty in the S-S and S-A sessions. We adapted the Greenhouse-Geisser correction of the degree of freedom for these cases.

**Table 1:** Experiment 1: Mean (SD) response times in milliseconds for each combination of experimental factors.

Discrimination difficulty	Target	Test Set	Sessions		
			Sham-Sham	Sham-Anodal	Sham-Cathodal
Easy	Absent	Test 1	528.0 (71.6)	536.6 (94.6)	525.5 (62.6)
		Test 2	519.9 (66.2)	507.2 (76.8)	518.0 (64.8)
	Present	Test 1	482.5 (50.8)	506.4 (74.9)	494.1 (46.4)
		Test 2	479.1 (46.7)	482.6 (59.4)	485.1 (58.0)
Intermediate	Absent	Test 1	546.1 (66.5)	564.1 (90.9)	562.2 (73.4)
		Test 2	545.8 (82.6)	535.9 (76.4)	554.2 (83.3)
	Present	Test 1	503.0 (54.7)	520.4 (74.7)	511.5 (51.7)
		Test 2	497.8 (52.8)	487.4 (53.9)	499.2 (52.6)
Difficult	Absent	Test 1	573.4 (76.7)	582.1 (106.4)	578.3 (57.4)
		Test 2	566.2 (86.9)	563.2 (64.4)	572.4 (79.4)
	Present	Test 1	514.6 (51.5)	532.8 (87.2)	526.2 (67.0)
		Test 2	501.6 (49.0)	506.6 (74.9)	509.2 (56.2)

The three repeated-measures ANOVAs showed a significant tDCS effect on search RTs in the S-A (anodal) session but not in the S-C (cathodal) and S-S (sham) sessions. That is, the main effect of the test-set factor was significant in the S-A session (*F*_1, 11_ = 11.19, *MSE* = 25394.79, *p* = .007, partial eta-squared [*η*_*p*_^2^] = .50), suggesting that the mean RT of Test 2 was significantly shorter than that of Test 1 due to the use of anodal tDCS in Test 2. The main effect of the test-set factor in the S-S and S-C sessions was not significant, indicating no significant difference in RTs between Tests 1 and 2 in these sessions: S-S (*F*_1, 11_ = .76, *MSE* = 1384.35, *p* = .402, *η*_*p*_^2^ = .07) and S-C (*F*_1, 11_ = 1.52, *MSE* = 3574.87, *p* = .243, *η*_*p*_^2^ = .12).

The results of ANOVAs further showed that the main effects of target and discrimination difficulty factors on the mean RTs, and the interaction between them, were significant in all three sessions. Participants responded faster when the target was present than when it was absent in all sessions: S-S (*F*_1, 11_ = 30.36, *MSE* = 90456.64, *p* < .001, *η*_*p*_^2^ = .73), S-A (*F*_1, 11_ = 35.56, *MSE* = 63882.54, *p* < .001, *η*_*p*_^2^ = .76), and S-C (*F*_1, 11_ = 58.41, *MSE* = 81415.29, *p* < .001, *η*_*p*_^2^ = .84). The participants also responded faster when the task was relatively easy than when it was difficult in all sessions: S-S (*F*_1.4, 15.1_ = 31.41, *MSE* = 23463.3, *p* < .001, *η*_*p*_^2^ = .74), S-A (*F*_1.3, 14.6_ = 27.75, *MSE* = 26147.6, *p* < .001, *η*_*p*_^2^ = .72), and S-C (*F*_2, 22_ = 57.28, *MSE* = 20529.58, *p* < .001, *η*_*p*_^2^ = .84). The target and discrimination difficulty factors significantly interacted with each other: The effect of target presence/absence was larger in the difficult search task than in the easy task in all three sessions: S-S (*F*_2, 22_ = 5.37, *MSE* = 1222.73, *p* = .013, *η*_*p*_^2^ = .33), S-A (*F*_2, 22_ = 8.90, *MSE* = 2100.98, *p* = .002, *η*_*p*_^2^ = .45), and S-C (*F*_2, 22_ = 5.25, *MSE* = 2188.57, *p* = .014, *η*_*p*_^2^ = .32). These results replicated the basic findings in typical visual search experiments [12, 13].

**Fig 2:**
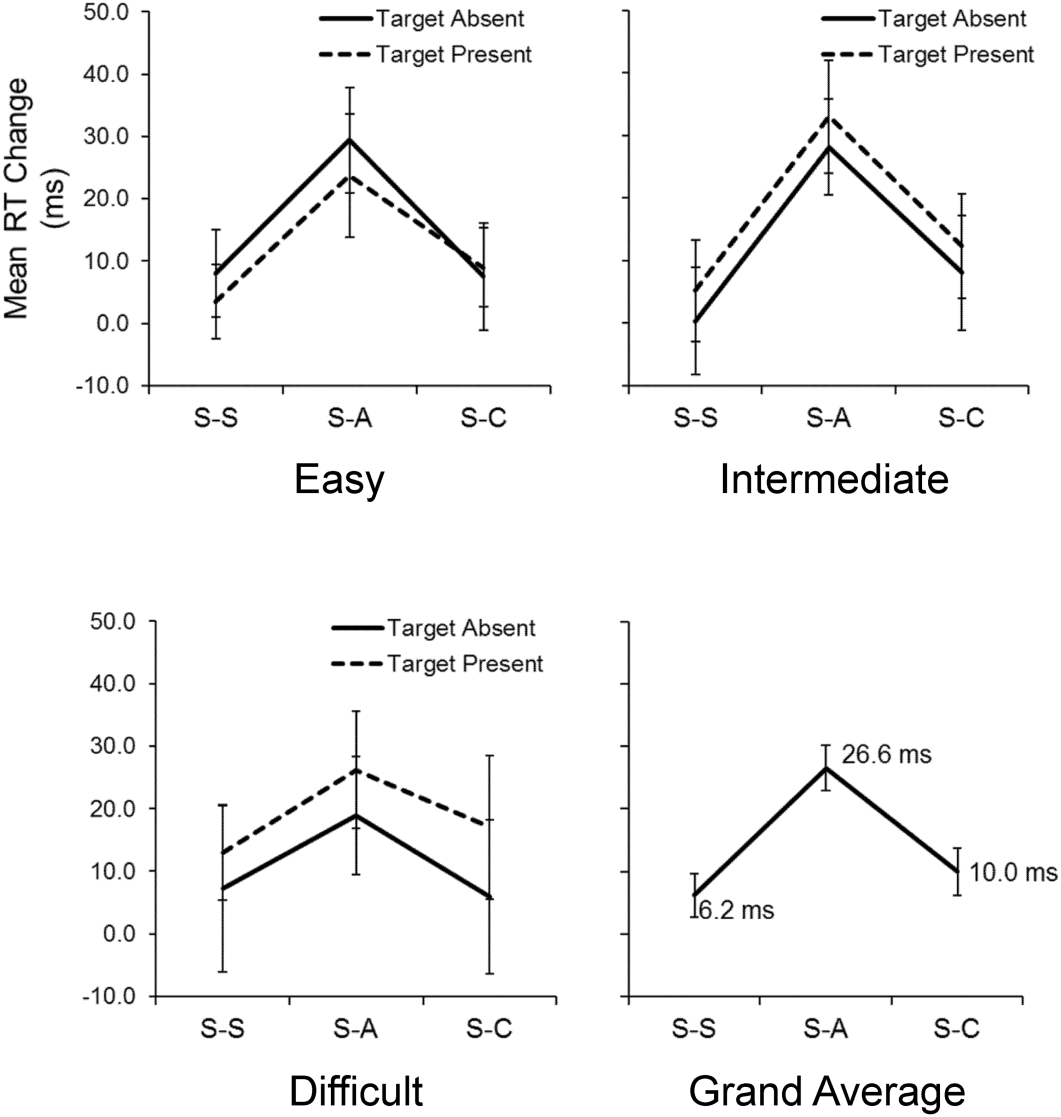
Experiment 1: Mean RT changes (Test 1 – Test 2) in the three sessions. The top and bottom left panels are the three discrimination difficulty levels (easy, intermediate, and difficult). The bottom right panel is the grand averages of RT changes after collapsing all task factor levels. The error bars indicate the standard error of the mean (SEM). S-S, Sham-Sham; S-A, Sham-Anodal; S-C, Sham-Cathodal.

Importantly, we found no significant two- or three-way interaction effect between the test-set and other task factors in the S-A session, indicating that the effect of anodal stimulation on search RT was not affected by the discrimination difficulty or the presence/absence of a target. Fig 2 shows the mean RT changes between Test 1 and Test 2 in the three sessions for each combination of factors, along with the grand averages after collapsing all task factor levels. On average, anodal stimulation improved mean RTs by 26.6 ms. Although the RT change between Test 1 and Test 2 of the S-C session was not statistically significant, there did seem to be a small trend: the change in RT (10.0 ms) of the S-C session was slightly greater than that of the S-S session (6.2 ms).

#### Subjective ratings of stimulation and individual performance

The participants’ ratings on subjective awareness of electrical stimulation after each visual search test are presented in the S1 Table. Overall, the mean ratings suggest that participants may have been aware of the differences between sham and active stimulation in the active sessions (3.8 vs. 8.2 for S-A and 5.6 vs. 7.3 for S-C). However, there was large individual variation in ratings, depending on session order. We further examined this variability.

First, we identified six participants who had high confidence (≥7) that they had received active electrical stimulation during either of the two tests of the S-S session (i.e., false alarms). They were participants 1, 3, 4, 8, 9, and 10 (S1 Table). Interestingly, all four participants who had the S-S session first (1, 3, 9, and 10) were in this group. This observation suggested that participants may have built up certain expectations of electrical stimulation before they entered the study, and rated their experience based not entirely on their actual sensations but at least partially on their prior expectations. Among the remaining six participants (2, 5, 6, 7, 11, and 12) who correctly judged the S-S session as a sham, only two or three (2, 7, and possibly 12) seemed able to confidently judge the differences between Tests 1 and 2 in the two active sessions (S-A and S-C).

**Fig 3:**
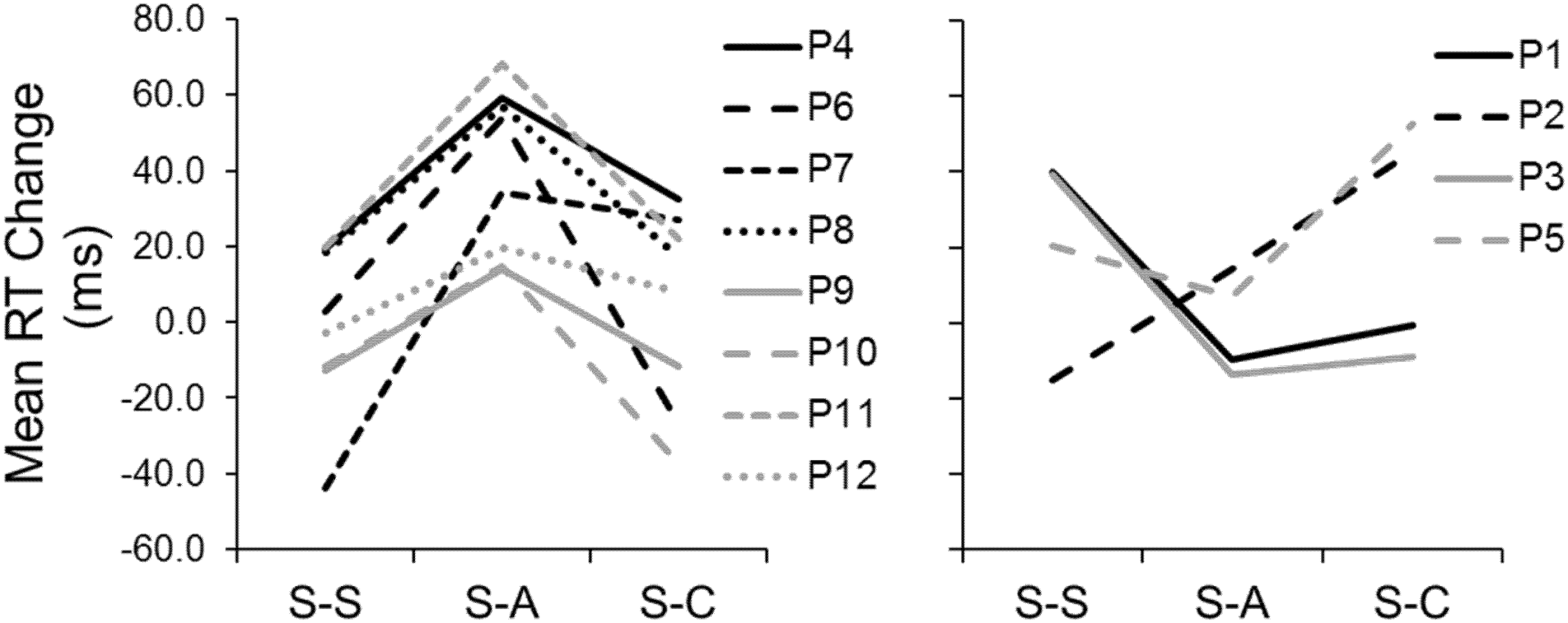
Experiment 1: Individual mean RT changes (Test 1 – Test 2) in the three sessions after collapsing other task factor levels. Left panel: Participants who had their greatest mean RT improvement during the S-A session. Right panel: Participants who had their greatest mean RT improvement during the S-S or S-C session.

Next, we examined whether those two or three participants who could differentiate between sham and active stimulation drove the significant effect of anodal tDCS in Experiment 1. We grouped all participants into two subgroups based on their RT changes between Tests 1 and 2 in the three sessions: participants who showed their largest RT improvement in the S-A session (Fig 3: left) and those who did not (Fig 3: right). The latter group includes some participants who could accurately differentiate sham from active stimulation (e.g., participant 2) and some who could not (e.g., participant 3). These results indicate that participants who were able to tell sham from active stimulation were not the main reason that we observed significant RT changes in the S-A session.

Also, we examined the ratings of all participants in the S-A session regardless of ratings in other sessions. We found that participants 1, 2, 3, 4, 7, 8, and 11 were relatively confident of no electrical stimulation in Test 1 (rating ≤6) but of electrical stimulation in Test 2 (rating ≥7). However, about half of these participants (participants 1, 2, and 3) did not show any RT improvement due to anodal stimulation (Fig 3: right panel), another indication that awareness of electrical stimulation was not the main reason for the significant RT change in the S-A sessions.

### Discussion of Experiment 1

We found that anodal tDCS did significantly improve visual search RT; cathodal tDCS produced a trend toward improvement, although it was not statistically significant. We also found the expected effects of factor manipulation: The significant main and interaction effects of target-distractor discrimination difficulty and target presence factors replicated the findings of previous studies [12, 13, 26]. However, the effects of anodal tDCS did not interact with any of the main search task factors (task difficulty and target presence/absence). The possible failure of single-blind manipulation in some participants could not explain these findings. We therefore conclude from Experiment 1 that anodal tDCS did not have any effect on the second stage of visual search (i.e., the attentive stage).

## Experiment 2

### Materials and methods

#### Rationale and design

In Experiment 1, we found an effect of tDCS on visual search times. We also found that search task factors known to affect the second stage of visual search models did not interact with the tDCS effect. In Experiment 2, we examined whether a factor known to affect the first stage in the visual search task, stimulus brightness manipulation, would interact with the tDCS effect. Also, to replicate our results of Experiment 1 and confirm the expected nonsignificant interaction between stimulus brightness and other factors affecting the second stage of the visual search, we continued manipulating the stage-two factors of discrimination difficulty and target presence/absence.

We did make one minor adjustment of the experimental design used in Experiment 1, which was to use two levels of discrimination difficulty in Experiment 2, rather than three levels. This modification helped us maintain a similar duration of tDCS stimulation across the two experiments by having the same number of task trials for the search test. Therefore, the experimental design of each session (S-S, S-A, or S-C) in Experiment 2 was a 2 (discrimination difficulty) × 2 (target presence) × 2 (test set) × 2 (stimulus brightness) repeated-measures factorial design. As before, the existence of any tDCS effect on RT would be revealed by a statistically significant test-set factor in each session.

#### Participants

By the end of Experiment 2, a total of 20 participants had been recruited. We initially recruited 18 participants to have three participants for each of the six counterbalanced session orders. However, we found that one participant could not finish all three required sessions, and one participant did not follow the experimental instruction on making speeded responses, producing more than 30% of RT outliers and errors. These two participants in the initial group were excluded from the study, and two more participants were recruited to keep the same number of subjects in each session order. Of the 18 subjects included in the analyses, nine were women. The mean (SD) of ages was 26.1 (3.9) years. All subjects reported normal or corrected-to-normal vision and all were right-handed.

As in Experiment 1, all participants gave written informed consent to participate. As part of the consent, participants were informed that they might receive electrical stimulation during the three experimental sessions, but they were not informed of when they might get such stimulation (making this again a single-blind design).

As in Experiment 1, all participants were paid for their participation. The study and consent procedures were approved by the Johns Hopkins Medicine Institutional Review Board.

#### Visual search task, apparatus, and stimuli

**Fig 4:**
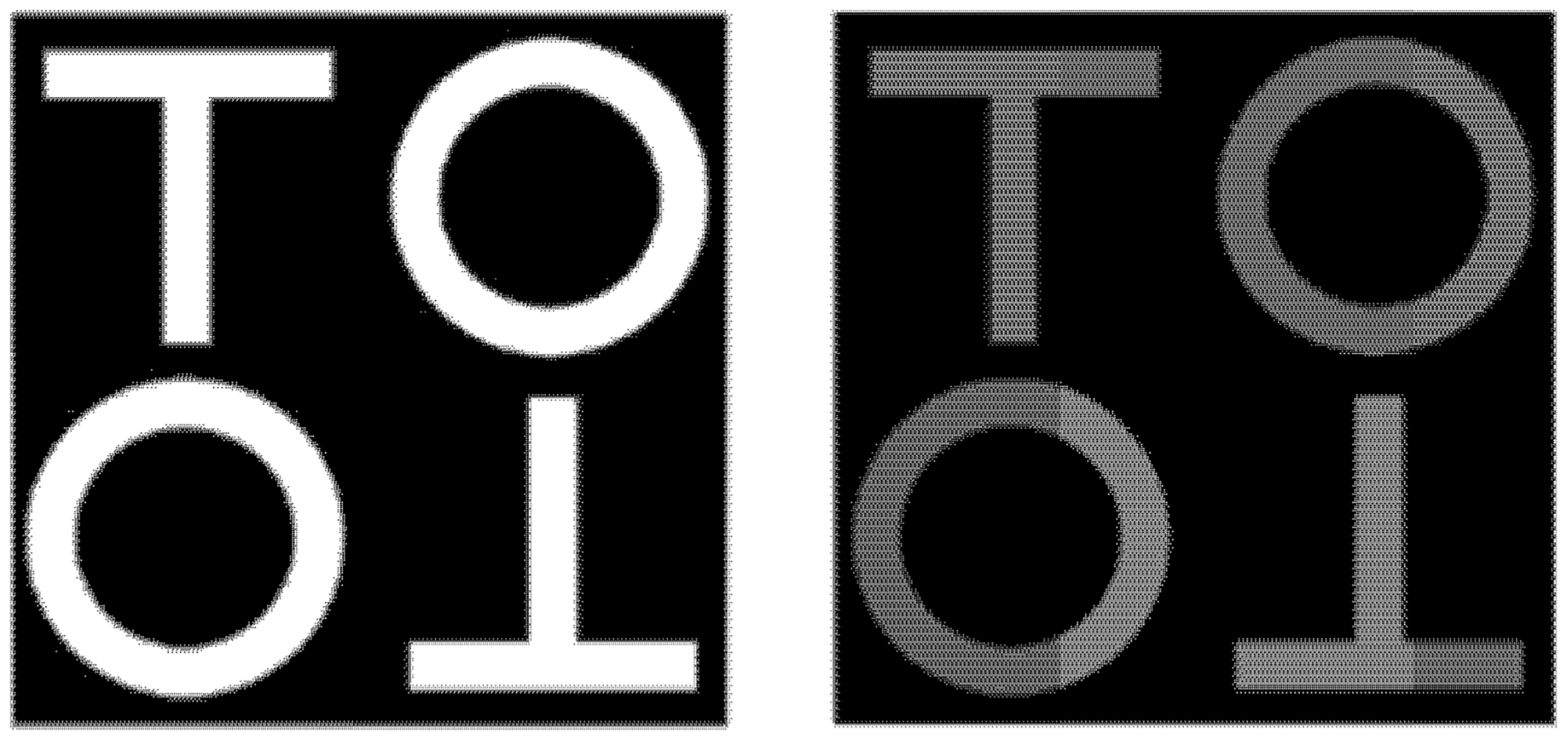
Experiment 2: Two examples of target-absent condition display. Left: Easy search condition with bright stimuli. Right: Easy search condition with dim stimuli. In the target-present condition, one of the upright “Ts” or “Ts” rotated 180° was replaced with a target “L.” As in Experiment 1, the search difficulty was manipulated by replacing two “Os” with “Ts” rotated by 90° and 270°.

The visual search task, apparatus, and stimuli of Experiment 2 were identical to those of Experiment 1, with one difference: We introduced the stimulus brightness manipulation (dim/bright) in Experiment 2. We reduced the grayscale value of white stimuli from 256 (135 cd/m^2^) to 80 (15 cd/m^2^) to create dim stimuli presented on a black background (0.3 cd/m^2^) (Fig 4).

#### Experimental procedure and direct current stimulation

Four blocks of trials, with 96 trials in each block, were conducted in each visual search test. All other experimental procedures and parameters of the tDCS application process, including the approximate duration of tDCS, were identical to those of Experiment 1.

### Analysis

#### Data cleaning

We removed all errorneous reponses, which constituted 3.3% (1,349 trials) of all responses made by participants. The same criteria for RT outliers as in Experiment 1 were used to filter out 2.0% of responses (822 trials) from all correct responses. All other parameters for the data cleaning procedure were the same as in Experiment 1.

#### Statistical analysis of RTs and subjective ratings

All the analytic procedures used on the results of Experiment 2, including three separate repeated-measures ANOVAs on mean RTs for the three tDCS sessions, a Bonferroni correction for multiple comparisons, and the descriptive analysis of subjective ratings, were the same as those used for Experiment 1.

### Results

#### Mean RTs and ANOVA

Table 2 shows the mean (SD) RTs for each combination of factors in Experiment 2. Repeated-measures ANOVAs showed that the results of Experiment 2 almost exactly replicated the findings of Experiment 1. As in Experiment 1, we found a significant main effect of the test-set factor in the S-A (anodal) session, which was an indicator of the significant anodal tDCS effect: Participants were faster in Test 2 than in Test 1 (*F*_1, 17_ = 13.75, *MSE* = 17995.89, *p* = .002, *η*_*p*_^2^ = .45). Interestingly, unlike the result we saw in Experiment 1, there was a significant main effect of the test-set factor in the S-C (cathodal) session in Experiment 2. Participants responded faster in Test 2 than in Test 1 due to the use of cathodal stimulation in Test 2 (*F*_1, 17_ = 14.83, *MSE* = 12912.25, *p* = .001, *η*_*p*_^2^ = .47). There was no significant effect of the test-set factor in the S-S (sham) session (*F*_1, 17_ = 1.42, *MSE* = 2872.31, *p* = .25, *η*_*p*_^2^ = .08).

**Table 2:** Experiment 2: Mean (SD) response times in milliseconds for each combination of experimental factors.

Discrimination difficulty	Target	Bright-ness	Test Set	Sessions		
				Sham-Sham	Sham-Anodal	Sham-Cathodal
Easy	Absent	Bright	Test 1	471.7 (48.7)	485.2 (45.2)	476.1 (61.6)
			Test 2	469.0 (49.6)	470.4 (50.2)	470.5 (48.5)
		Dim	Test 1	509.8 (46.0)	515.9 (50.7)	511.1 (58.3)
			Test 2	495.8 (43.7)	495.1 (47.5)	497.8 (46.3)
	Present	Bright	Test 1	470.0 (50.8)	477.3 (65.6)	469.8 (54.6)
			Test 2	464.6 (54.3)	466.1 (62.4)	461.1 (52.6)
		Dim	Test 1	500.3 (63.0)	509.1 (67.0)	506.2 (64.1)
			Test 2	500.4 (61.8)	495.5 (62.9)	484.3 (58.6)
Difficult	Absent	Bright	Test 1	523.9 (65.0)	534.2 (68.0)	529.3 (62.7)
			Test 2	509.6 (62.4)	517.4 (64.7)	512.6 (73.3)
		Dim	Test 1	558.3 (61.1)	568.4 (64.6)	561.3 (68.0)
			Test 2	550.2 (62.2)	557.1 (62.8)	546.9 (64.2)
	Present	Bright	Test 1	489.9 (61.6)	494.6 (66.8)	485.1 (71.0)
			Test 2	489.2 (59.3)	480.5 (65.5)	474.7 (64.6)
		Dim	Test 1	518.5 (64.0)	529.5 (66.2)	520.0 (67.6)
			Test 2	513.0 (60.1)	505.7 (53.0)	504.1 (64.3)

As in Experiment 1, results also showed that participants were faster when the target was present than when it was absent in all three sessions: S-S (*F*_1, 17_ = 8.78, *MSE* = 22782.59, *p* = .009, *η*_*p*_^2^ = .34), S-A (*F*_1, 17_ = 14.65, *MSE* = 38648.95, *p* = .001, *η*_*p*_^2^ = .45), and S-C (*F*_1, 17_ = 28.69, *MSE* = 45142.11, *p* < .001, *η*_*p*_^2^ = .63). The participants also responded faster when the task was relatively easy than when it was difficult in all sessions: S-S (*F*_1, 17_ = 100.64, *MSE* = 82547.97, *p* < .001, *η*_*p*_^2^ = .86), S-A (*F*_1, 17_ = 108.40, *MSE* = 83735.96, *p* < .001, *η*_*p*_^2^ = .86), and S-C (*F*_1, 17_ = 57.98, *MSE* = 74423.39, *p* < .001, *η*_*p*_^2^ = .77). The two-way interaction of discrimination difficulty and target factors was also significant in all three sessions, as in Experiment 1: S-S (*F*_1, 17_ = 24.36, *MSE* = 16279.60, *p* < .001, *η*_*p*_^2^ = .59), S-A (*F*_1, 17_ = 28.03, *MSE* = 24686.05, *p* < .001, *η*_*p*_^2^ = .62), and S-C (*F*_1, 17_ = 40.34, *MSE* = 19609.26, *p* < .001, *η*_*p*_^2^ = .70).

There was a significant main effect of the stimulus brightness manipulation in all three sessions. Participants responded faster to bright stimuli than to dim stimuli: S-S (*F*_1, 17_ = 624.46, *MSE* = 75123.99, *p* < .001, *η*_*p*_^2^ = .97), S-A (*F*_1, 17_ = 269.03, *MSE* = 70778.27, *p* < .001, *η*_*p*_^2^ = .94), and S-C (*F*_1, 17_ = 232.04, *MSE* = 71766.19, *p* < .001, *η*_*p*_^2^ = .93). As expected, neither the discrimination difficulty nor the target factor interacted with the brightness manipulation in any of the three tDCS sessions.

Importantly, we found no significant interaction between the test-set and target/difficulty factors in the S-A and S-C sessions. Interestingly, the test-set factor also did not interact with the stimulus brightness factor in these active tDCS sessions. That is, the main effects of anodal and cathodal tDCS were completely independent of the effect of all search task factors, including stimulus brightness.

**Fig 5:**
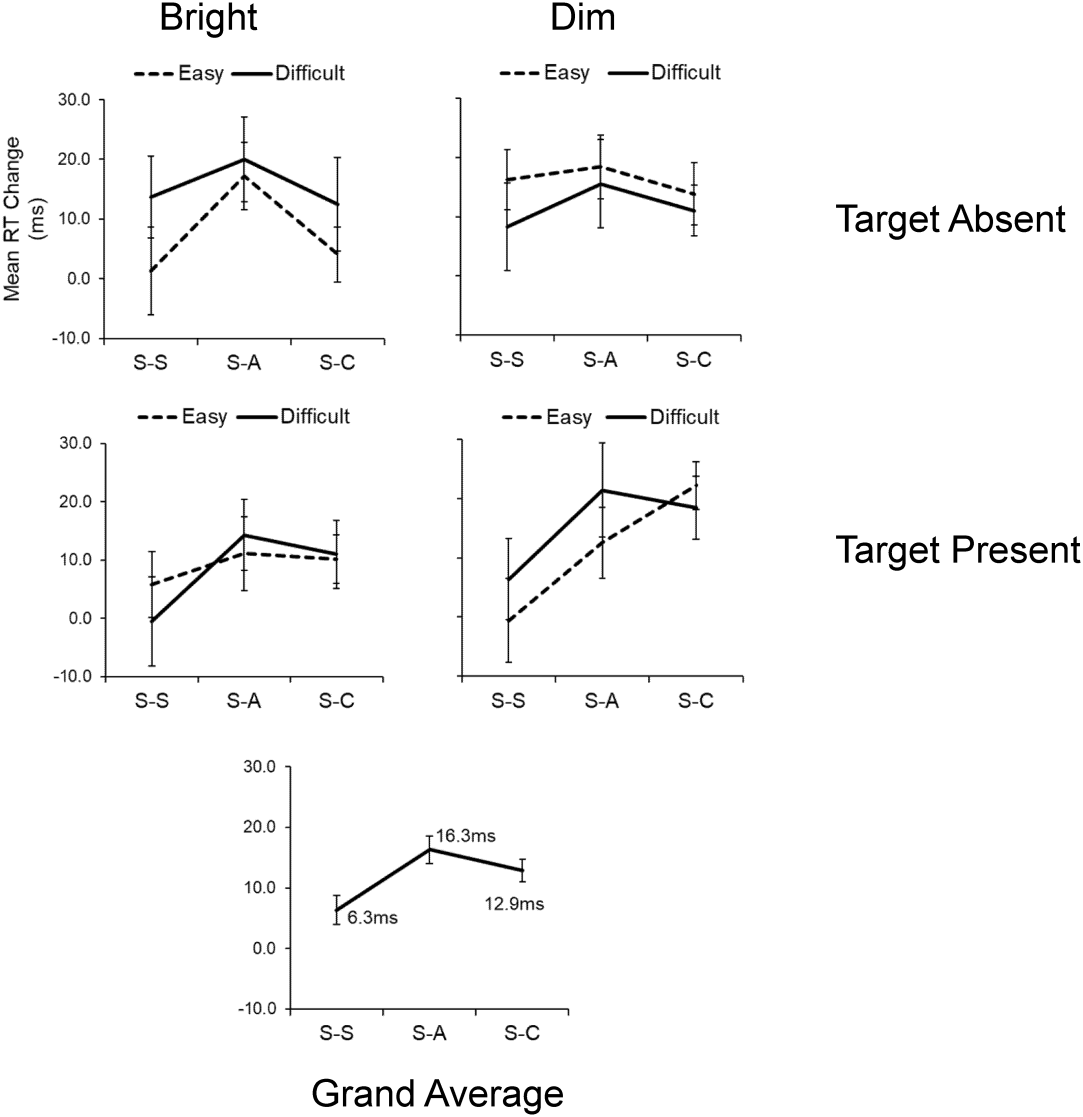
Experiment 2: Mean RT change (Test 1 – Test 2) in the three tDCS sessions. Top and middle panels are arranged by the combination of stimulus brightness (bright/dim) and target (absent/present) factors. The bottom panel shows the grand averages of RT change after collapsing all task factor levels. The error bars indicate SEM.

**Fig 6:**
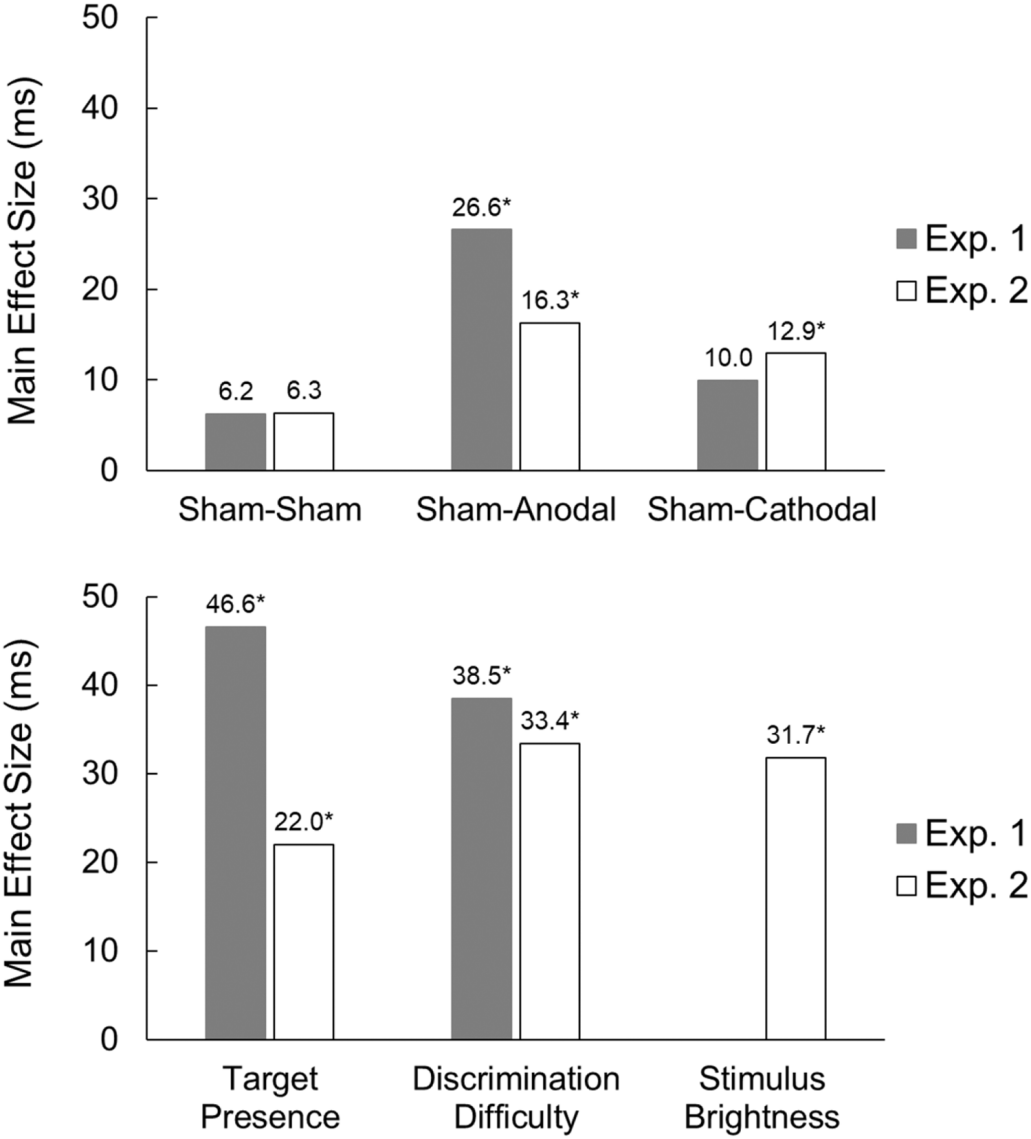
Summary of the main effect sizes of Experiments 1 and 2. Top panel: effect sizes of tDCS. Bottom panel: effect sizes of task factors. Effect sizes (in milliseconds) were calculated between the levels of a task factor (e.g., discrimination difficulty effect size = mean difficult search RT−mean easy search RT) after collapsing all other task factor levels. Note that the stimulus brightness factor was manipulated only in Experiment 2. *p < .017.

Fig 5 shows the mean RT changes from Test 1 to Test 2 in all combinations of experimental factors, along with the grand averages after all factor levels were collapsed. On average, the anodal and cathodal tDCS improved mean RT from Test 1 to Test 2 by 16.3 ms and 12.9 ms, respectively. The mean RT change from Test 1 to Test 2 in the S-S session was 6.3 ms, which was almost identical to that of the S-S session in Experiment 1 (see Fig 6 for a summary of the main effect sizes of Experiments 1 and 2).

#### Subjective ratings on stimulation and individual performance

The ratings of perceived electrical stimulation by all participants in Experiment 2 are presented in supporting information (S2 Table). As in Experiment 1, we first identified those who believed with high confidence (rating ≥7) that they were receiving electrical stimulation in at least one of the two search tests of the S-S session. They were participants 2, 4, 5, 8, 10, 14, and 15. Similar to Experiment 1 participants, all six participants (2, 5, 8, 10, 14, and 15) who completed the S-S session first felt that they were receiving electrical stimulation. Those who did the active stimulation session first (S-A or S-C) tended to correctly judge that they did not receive electrical stimulation in the S-S session.

**Fig 7:**
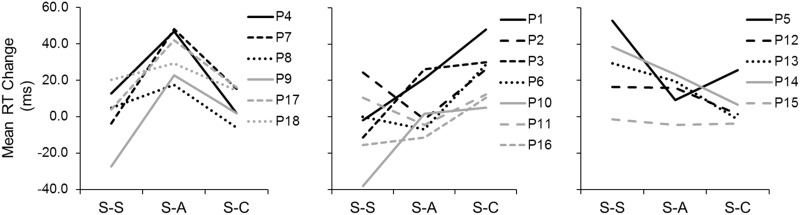
Experiment 2: Individual mean RT changes (Test 1 – Test 2) in the three sessions after collapsing other task factor levels. Left panel: Participants who had their greatest mean RT improvement during the S-A session. Middle panel: Participants who had their greatest mean RT improvement during the S-C session. Right panel: Participants who had their greatest mean RT improvement during the S-S session.

To investigate whether the observed anodal and cathodal tDCS effects correlated with participants’ sensations of electrical stimulation, we compared the confidence ratings in the S-A and S-C sessions with RT improvement in these sessions for all participants (Fig 7). Eleven participants (2, 3, 4, 5, 6, 8, 11, 14, 15, 17, and 18) correctly judged that they were not receiving electrical stimulation in Test 1 (rating ≤6) but they were in Test 2 (rating ≥7) of the S-A session. Less than half of the 11 participants (4, 8, 17, and 18) actually showed the largest RT improvement in the S-A session (Fig 7: left). Eleven participants (1, 2, 4, 5, 6, 8, 11, 13, 14, 15, and 18) judged correctly with high confidence that they were not receiving stimulation in Test 1 but they were in Test 2 of the S-C session. Again, less than half of them (1, 2, 6, and 11) showed the largest RT improvement from Test 1 to Test 2 in the S-C session (Fig 7: middle).

We interpret these results as showing that many participants were not completely blind to the electrical stimulation when active stimulation immediately followed a sham. However, as in Experiment 1, we concluded that the subjective sensation of electrical stimulation by itself, and its possible effect on search performance, could not explain the significant effects of anodal and cathodal tDCS in Experiment 2.

### Discussion of Experiment 2

Experiment 2 replicated the significant effect of anodal tDCS found in Experiment 1, which improved RTs. Moreover, the trend for cathodal tDCS effect on search RTs found in Experiment 1 became a statistically significant effect in Experiment 2. Interestingly, the direction of the cathodal tDCS effect on RT was the opposite of our expectations: It shortened, rather than prolonged, the visual search RTs (Fig 6).

The results of Experiment 2 almost exactly replicated the results of Experiment 1 regarding the effects of the behavioral task factors (discrimination difficulty and target presence/absence) thought to affect the second stage of visual search. In addition, Experiment 2 also showed the expected significant effect of stimulus brightness, a factor known to have an effect on the first stage of visual search. The interaction patterns of these factors followed the patterns expected from the two-stage model for visual search and prior experimental studies.

However, there was no evidence of an interaction between active tDCS (anodal and cathodal) and any of these task factors in Experiment 2. These results suggest that tDCS is not producing its effects on RTs at either the first or the second stage of processing involved in the visual search task.

## Experiment 3

### Materials and methods

#### Rationale and design

Experiments 1 and 2 could be interpreted as showing that tDCS over the visual cortex (Oz) significantly improved visual search RTs, whether it was anodal or cathodal stimulation. One potential concern about this interpretion is that, because the common reference electrode site (Cz) is near the motor cortex, motor effects of the tDCS could have facilitated the key pressing responses and resulting RTs in Experiments 1 and 2. For example, the observed RT improvement in the S-A session of Experiment 1 could have been the outcome of the joint contribution of the anodal stimulation over Oz and cathodal stimulation over Cz. Given that cathodal tDCS might produce a similar effect on neuronal activities as anodal tDCS produced (i.e., the result of Experiment 2), this is a reasonable concern.

To test this possibility, we conducted a single-session control experiment similar to the S-A session of Experiment 1 because this was the session in which the largest tDCS effect size was observed (Fig 6). In Experiment 3, we kept the cathodal electrode over Cz, but placed the anodal electrode over an extracephalic location, the right upper arm (bicep), instead of Oz. If the claimed effect of anodal tDCS over Oz in the S-A session of Experiment 1 or 2 was mainly determined by the stimulation of motor regions through cathodal tDCS over Cz, then we should observe similar effects regardless of the location of the anodal electrode. The absence of such an effect would help confirm that the observed tDCS effect of Experiments 1 and 2 was indeed due to the stimulation of the visual cortex.

There were two identical search tests in Experiment 3: One test was sham, and the other was tDCS (anodal over bicep and cathodal over Cz). There were also three levels of task difficulty factor (easy, intermediate, and difficult) and two levels of target factor (presence and absence). Thus, the experimental design was 3 (discrimination difficulty) × 2 (target presence and absence) × 2 (sham-tDCS) repeated-measures factorial design. A significant search RT difference between sham and tDCS would indicate the effect of cathodal tDCS over Cz.

#### Participants

There were 16 healthy participants in Experiment 3, of which 10 were women. The mean (SD) of ages was 22.9 (2.6) years. All participants reported normal or corrected-to-normal vision, and all were right-handed. All participants gave written informed consent to participate. As part of the consent, participants were informed that they might receive electrical stimulation during the experiment, but they were not informed of when they might get such stimulation (a single-blind design).

All participants were paid for their participation. The study and consent procedures were approved by the Johns Hopkins Medicine Institutional Review Board.

#### Experimental task, apparatus, procedure, and direct current stimulation

The experimental task, apparatus, and procedure of Experiment 3 were identical to those of the S-A session of Experiment 1 except for the counterbalance of the order of sham and tDCS tests. Half of the participants were randomly selected to receive the sham test first and then the tDCS test. The other half of the participants received the tDCS test first and then the sham test. We counterbalanced the order of sham and tDCS tests to minimize the practice effect across two search tests in a single-session experiment. The time break between sham and anodal tests was 30 minutes. All the parameters for tDCS stimulation, including the average duration of stimulation, were the same as those of Experiment 1 except for the extracephalic location of the anodal electrode.

### Analysis

#### Data cleaning and statistical analysis

We removed all errorneous responses, which constitute 3.2% (366 trials) of all responses. The same criteria for RT outliers used in Experiment 1 were used to remove 3.7% of responses (426 trials) from all correct responses. We performed a single repeated-measures ANOVA on mean RTs of individuals’ data at a significance level of .05. Two post hoc *t* tests were performed to determine whether the counterbalancing of tDCS and sham order had any effect on RT changes, with a family-wise error rate for multiple comparisons controlled at .025 (.05/2) with a Bonferroni correction.

### Results

Table 3 shows the mean (SD) of search RTs for each combination of factors in Experiment 3. The test of the equality of variance (Mauchly’s test) showed no violation of sphericity assumption for covariance matrix in any statistical tests. The repeated-measures ANOVA showed that the main effect of the sham-tDCS factor was not significant (*F*_1, 15_ = .10, *MSE* = 67.69, *p* = .92, *η*_*p*_^2^ = .001). That is, the RT difference of 4.8 ms between the sham (573.0 ms) and tDCS (568.2 ms) tests was not statistically different from 0. As in Experiment 1, the main effect of the task difficulty factor was significant, suggesting longer mean RT in the difficult search than in the easy search (*F*_2, 30_ = 154.95, *MSE* = 33519.35, *p* < .001, *η*_*p*_^2^ = .91). Also, the mean RT for the target-present search was significantly shorter than the mean RT for the target-absent search (*F*_1,11_ = 40.31, *MSE* = 143510.94, *p* < .001, *η*_*p*_^2^ = .73). We did not find any significant interaction between the sham-tDCS, task difficulty, and target factors.

**Table 3:** Experiment 3: Mean (SD) response times in milliseconds for each combination of experimental factors.

Discrimination difficulty	Target	Sham-tDCS	
		Sham	tDCS
Easy	Present	569.3 (70.4)	574.2 (70.5)
	Absent	515.4 (66.2)	525.1 (58.0)
Intermediate	Present	597.9 (68.1)	600.5 (71.8)
	Absent	543.0 (67.5)	545.8 (61.8)
Difficult	Present	615.3 (69.2)	625.3 (78.6)
	Absent	562.8 (67.2)	559.6 (63.8)

Post hoc comparisons (equal variance assumed; two-tailed) showed that the mean RT of those who received the sham first in the first search test was not significantly different from the mean RT of those who received tDCS first in the first search test (*t*_14_ = −1.26, *p* = .23). Also, the mean RT of those who received the sham in the second test was not different from the mean RT of those who received tDCS in the second test (*t*_14_ = −.99, *p* = .34). Overall, these results confirmed that the cathodal stimulation of motor cortex with an anodal electrode over an extracephalic location did not have any effect on visual search RT.

### Discussion of Experiment 3

Experiment 3 was conducted because of a concern that the effects of tDCS observed in Experiments 1 and 2 may have been confounded or entirely caused by the unintended stimulation of motor regions subjacent to Cz. This concern was reasonable because cathodal tDCS seemed to have the same direction of effect as did anodal tDCS, especially in Experiment 2. Experiment 3 was completed to identify a possible contribution of pure motor stimulation to our findings. Experiment 3 did not show any effect of tDCS on search RTs with cathodal stimulation at Cz and with the anodal electrode over an extracephalic location. This is direct evidence that motor effects from Cz stimulation were not a cause in the results of Experiments 1 and 2.

In addition, the results of Experiments 1 and 2 and the results of previous studies already suggest that the possible contribution of motor cortex stimulation in our study is not a critical issue. In Experiments 1 and 2, anodal stimulation over Cz (i.e., the sham-cathodal sessions of Experiments 1 and 2) had no significant effect on RT (Experiment 1) or produced a small effect compared to cathodal stimulation over Cz (Experiment 2). These results are almost the opposite of those of previous studies that focused on the effects of direct stimulation of the motor cortex with tDCS [63, 64]. In those previous studies, anodal tDCS over the motor cortex improved motor responses or enhanced the excitability of motor neurons, while cathodal tDCS either had no effect on motor responses or generated an effect opposite that of anodal tDCS.

We believe that our study results and previous results strongly support the inference that the effects of tDCS in Experiments 1 and 2 were indeed due to tDCS-induced modulation of the visual cortex. It is very unlikely that incidental stimulation of the motor cortex confounded the results of our experiments.

## General discussion

We believe that these results are noteworthy because of several major findings and their implications for the functional mechanism of tDCS.

### tDCS effects

One of the major findings of our study was a significant effect of tDCS on visual search RTs, which was replicated across two experiments. The effect of anodal tDCS over the visual cortex was significant in both experiments. There was a similar but weak trend of cathodal tDCS effect on search RTs in Experiment 1. Cathodal tDCS generated a statistically significant effect in Experiment 2, although its effect size was smaller than that of anodal tDCS. The finding that tDCS had a significant, replicable effect on visual search times adds to the evidence that tDCS can modulate mental processes when applied during the actual processing (“online” stimulation). Moreover, it supports the notion that these effects are independent of any placebo effects of tDCS stimulation.

Of course, the fact that both anodal and cathodal tDCS had facilitatory effects on search RTs, rather than opposite effects, is somewhat puzzling, although not unprecedented [65–67]. This interesting finding suggests that even if cathodal tDCS has an inhibitory effect on underlying neurophysiological processes, the complexity of neuronal information processing makes it difficult to predict the behavioral effects of tDCS. Also, studies have shown that the magnitude and direction of tDCS effects could be very sensitive to the particular experimental parameters and variants in tDCS protocols, such as task types, stimulation duration and intensity, electrode placement montage, population type (clinical vs. healthy individuals), and individual differences [60, 68–70]. One possible explanation for the counterintuitive effects of cathodal tDCS may be the large individual differences that we found, which are discussed below.

### The locus of tDCS effect in the two-stage model of visual search

Experiments 1 and 2, taken together, showed that stimulus brightness, discrimination difficulty, and the presence/absence of the target significantly affected search RTs in the search task (see Fig 6), replicating effects across the experiments and following the expected pattern of results cited in the literature. These findings further validate our investigative rationale and experimental designs intended to probe the origin(s) of the tDCS effect.

However, there was no interaction effect between tDCS and task factors, which we consider a major finding in our study. In keeping with additive factor logic, this means that neither anodal nor cathodal tDCS over the occipital region affected either of the two processing stages thought to be central to performance on the visual search task, the feature extraction and the attentive target comparison stages.

The replication of results across two experiments helps rule out weak statistical power as a possible explanation for not observing an interaction between task factors and tDCS. Both of our experiments reliably replicated the existence and nonexistence of interactions between behavioral task factors that were expected based on numerous prior studies.

We suggest that additive factor logic and its extension [28–30, 33] help us to infer how tDCS is exerting its effect on RTs in the visual search task we used. Recall that, given the significant main effect of tDCS, two main assumptions were required for a prediction of interaction pattern between search task factors and tDCS. One is that the relevant processing stages are organized as a serial-parallel network [12, 19]. The other assumption is that tDCS and other experimental factors selectively influence specific processes within the serial-parallel network.

Since tDCS did not interact with the task difficulty or stimulus brightness factors at all in our two experiments, we now have two possible logical conclusions. One is that the serial-parallel network is not the correct architecture of the search processes used in our task, and therefore additive factor logic does not apply as we expected. This would imply that tDCS was exerting its effects in our experiments on stages of processing not specified in the two-stage model, or that the visual search processing stages are much more complex than the two-stage model suggests. However, since every other experimental outcome with the task we used seems to fit well within the sequential two-stage model [18, 19], both of these possibilities seem very unlikely.

An explanation we consider highly probable is that tDCS does not selectively and exclusively affect any particular search processing stage as we anticipated. Instead, its effect is pervasive across different processing stages in a way that proportionally affects the duration of all processes without generating expected interaction effects with other experimental factors. Simply put, in this account the assumption of a selective influence of tDCS on visual search processes does not hold.

Interestingly, this logical conclusion has a matching theoretical concept that could help us understand this account of the tDCS effect in our study. There have been attempts by Dzhafarov and colleagues [31, 71] to extend the original notion of selective influence by Sternberg [28] to a situation where the process durations could be correlated. According to them, an individual process duration could be broken down into two independent components (or random variables): one unique to a process and the other shared with other processes. Applying this concept of dual-component variability for process duration to our study, we suggest that tDCS is an electrophysiological factor affecting the common component in all search processes, and the set of experimental factors (discrimination difficulty, target presence, and stimulus brightness) represents the behavioral factors selectively affecting the component unique to the process. In this conception, the effect of tDCS is a result of its diffuse facilitation of cortical processing associated with visual cortex. On the other hand, the effect of task factors is the result of altering the duration of the visual information processing by manipulating specific aspects of visual information (e.g., construction of individual feature and/or master saliency maps and the target identification in saliency maps). How this diffuse effect can be tested experimentally is not clear. Similar questions about the disassociation of two sources of variability, the systematic background activity across different domains, and the task-specific process components, have also been raised in a different context in a previous study [72].

With stimulating electrodes placed over Oz and Cz, we expected that maximal tDCS effects would be achieved in the cerebral tissue immediately below the electrode(s). While we did find a tDCS effect, we cannot readily make a neuroanatomical association because we did not find interactions with search task parameters that would have linked the tDCS effects to alterations in the first and/or second stage of the search task and, therefore, indirectly to the primary visual cortex and surrounding regions.

However, we must also consider the possibility that the tDCS effects we found are due to stimulation of the cerebral tissue between Oz and Cz, namely, the parietal cortices and especially the rPPC, a critical cortical region in top-down attentional control [73]. Given our explanation that the effects of tDCS may be spanning each of the two processing stages, the cortical areas affected by tDCS could be wider than we anticipated, including the rPPC.

Previous studies have shown conflicting results about the effect of tDCS over the rPPC during visual search. While some studies have shown that anodal tDCS stimulation over the rPPC had no effect on mean search RT and that cathodal tDCS slowed it [7, 8], one study [58] found that anodal tDCS shortened visual search RT compared to a sham condition (cathodal tDCS was not examined in this study). These conflicting results make it difficult for us to assess the possible effect of incidental rPPC stimulation on search RT. However, we do not believe there was a confounding effect of the rPPC in our study. If tDCS was, in fact, stimulating the rPPC and thus affecting the search RT through the top-down attentional control process, we would have been more likely to find a significant interaction between anodal/cathodal tDCS and the discrimination difficulty/target factors that selectively influence the attentive processing stage. We did not find any such interaction in our two experiments. Therefore, we believe that the disassociation of the effect of tDCS and behavioral task factors still holds, regardless of a conceivable effect from incidental stimulation of the rPPC.

Of course, this means that it is still unclear what neuroanatomical structures may be sites of the effects of tDCS. Given that our tDCS procedure and parameters could not help us identify specific regions of interest, answering this question may require a different tDCS protocol with a better focal targeting capability, such as high-definition tDCS [74, 75].

### Individual differences in tDCS effects

Our results suggest individual differences in the way participants responded to anodal and cathodal stimulation, although detecting such differences was not an *a priori* goal of our experiments. As shown in Fig 3 for Experiment 1, most individuals who had a significant change in RT due to anodal tDCS tended to respond only to anodal stimulation and not to cathodal stimulation. One exception was participant 7, who had significant changes in RT when stimulated with either anodal or cathodal tDCS. Although the sample size was small because of group split, a pairwise *t* test (equal variance assumed; two-tailed) on the RT changes in two sessions (S-A and S-C) shown in Fig 3 (left panel) supports this observation. In Fig 3 (left panel), the mean RT change was 40.1 ms in the S-A session versus 3.8 ms in the S-C session (*t*_7_ = 4.29, *p* < .01).

Similarly, in Experiment 2, when individuals were classified based on their RT improvement due to anodal and cathodal tDCS, it was almost always the case that an individual who showed a large RT improvement in one tDCS session did not show it in both sessions. That is, individuals with greater RT improvement in the S-A session tended not to show any RT improvement in the S-C session (Fig 7: left panel) and vice versa (Fig 7: middle panel). Two separate pairwise *t* tests (equal variance assumed; two-tailed) on the RT changes in the S-A and S-C sessions shown in Fig 7 (left and middle panels) seem to support this observation as well. In participants identified in Fig 7 (left panel), the mean RT change in the S-A session was greater than that in the S-C session (34.7 ms vs. 7.2 ms, *t*_5_ = 6.28, *p <* .01), whereas in participants identified in Fig 7 (middle panel), the mean RT change in the S-C session was greater than that in the S-A session (22.9 ms vs. 3.5 ms, *t*_6_ = 4.20, *p <* .01). One exception was participant 3 (P3; Fig 7: middle panel), who showed almost the same amount of RT change in both the S-C and S-A sessions (30.0 ms vs. 26.1 ms). (Participant 10 also showed almost the same amount of RT change in these two sessions, but the amount of change was practically zero.)

While we acknowledge that our observations on possible individual differences in participants’ responses to tDCS stimulation are tentative and may lack statistical power due to small sample size, these observations are in line with previous studies on individual variation in participants’ responses to tDCS stimulation [69, 76, 77]. Also, our observations on individual differences may explain, in part, why there was no significant effect of cathodal tDCS in Experiment 1: Experiment 1 participants’ response characteristics may have been accidentally biased toward anodal tDCS in the visual search tests due to a relatively small sample size compared to Experiment 2.

### Conclusion

Our experiments confirmed that tDCS over the visual cortex had a significant effect on RTs in an attention-demanding visual search task, bolstering the validity of tDCS effects. However, we also found that the effect of tDCS seemed to be quite disassociated from the way behavioral task factors affected visual search performance within the well-established theoretical framework for such processes. These results suggest the need for caution in interpreting the effect of tDCS on behavioral measures, as the underlying mental processes responsible for the behavioral outcomes may not have direct correspondences with the tDCS effects on neural tissues. Caution is particularly warranted when there are large individual differences in the neurophysiological responses to electrical stimulation.

## Supporting information

S1 File Experiment 1: Individual reaction time and error rate data. (XLSX)

S2 File Experiment 2: Individual reaction time and error rate data. (XLSX)

S3 File Experiment 3: Individual reaction time and error rate data. (XLSX)

S1 Table Experiment 1: Confidence ratings for tDCS stimulation (sorted by session order). (DOCX)

S2 Table Experiment 2: Confidence ratings for tDCS stimulation (sorted by session order). (DOCX)
