# Correction: Transcranial direct current stimulation (tDCS) facilitates overall visual search response times but does not interact with visual search task factors

**DOI:** 10.1371/journal.pone.0199565

**Published:** 2018-06-19

**Authors:** Kyongje Sung, Barry Gordon

There is an error in the Target column of Table 3. The labels “Present” and “Absent” should be reversed for each Discrimination difficulty. Please see the corrected Table 3 below.

**Table 3:** Experiment 3: Mean (SD) response times in milliseconds for each combination of experimental factors.

Discrimination difficulty	Target	Sham-tDCS	
		Sham	tDCS
Easy	Absent	569.3 (70.4)	574.2 (70.5)
	Present	515.4 (66.2)	525.1 (58.0)
Intermediate	Absent	597.9 (68.1)	600.5 (71.8)
	Present	543.0 (67.5)	545.8 (61.8)
Difficult	Absent	615.3 (69.2)	625.3 (78.6)
	Present	562.8 (67.2)	559.6 (63.8)

The values in the Target column in S3 File are labeled incorrectly. The legend should read: 1: Target present and 2: Target absent. Please see the correct S3 File below.

## Supporting information

S3 File Individual reaction time and error rate data. (XLSX)
