# Supplementary material for: Transcranial direct current stimulation (tDCS) facilitates overall visual search response times but does not interact with visual search task factors
Source: PLoS One. 2018 Mar 20;13(3):e0194640. doi: 10.1371/journal.pone.0194640 (PMC5860774; doi:10.1371/journal.pone.0194640)
Supplement: S1 Table — (DOCX) [file pone.0194640.s004.docx]

**Table S1. Experiment 1: Confidence ratings for tDCS stimulation (sorted by session order).**

| Participant | Session Order* | Sham-Sham | | Sham-Anodal | | Sham-Cathodal | |
| --- | --- | --- | --- | --- | --- | --- | --- |
|  |  | Test 1 | Test 2 | Test 1 | Test 2 | Test 1 | Test 2 |
| 6 | A-C-S | 4 | 6 | 5 | 5 | 5 | 5 |
| 11 | A-C-S | 4 | 5 | 2 | 8 | 5 | 5 |
| 4 | A-S-C | 2 | 8 | 5 | 9 | 9 | 9 |
| 8 | A-S-C | 7 | 2 | 2 | 8 | 8 | 5 |
| 7 | C-A-S | 2 | 4 | 0 | 10 | 0 | 7 |
| 12 | C-A-S | 4 | 6 | 1 | 5 | 1 | 10 |
| 2 | C-S-A | 0 | 0 | 0 | 10 | 5 | 10 |
| 5 | C-S-A | 2 | 5 | 10 | 10 | 10 | 10 |
| 1 | S-A-C | 5 | 10 | 0 | 10 | 5 | 10 |
| 9 | S-A-C | 5 | 8 | 10 | 5 | 10 | 5 |
| 3 | S-C-A | 10 | 0 | 1 | 10 | 0 | 1 |
| 10 | S-C-A | 10 | 10 | 10 | 8 | 9 | 10 |
| Mean  (SD) | | 4.6  (3.1) | 5.3  (3.4) | 3.8  (4.1) | 8.2  (2.1) | 5.6  (3.7) | 7.3  (3.0) |

^*^ A = Sham-Anodal, C = Sham-Cathodal, and S = Sham-Sham.
